# Incidence and risk factors for hospital-attributable central line-associated bloodstream infections in adult inpatients in a tertiary hospital

**DOI:** 10.1017/ash.2025.10149

**Published:** 2026-01-29

**Authors:** Shalvi Arora, Pinhong Jin, Aung Myat Oo, May Kyawt Aung, Edwin Philip Conceicao, Yong Yang, Jean Xiang Ying Sim, Molly Kue Bien How, Ismail Bin Sazali, Lai Chee Lee, Indumathi Venkatachalam, Moi Lin Ling

**Affiliations:** 1 Department of Infection Prevention and Epidemiology, Singapore General Hospital, Singapore, Singapore; 2 Department of Infectious Diseases, Singapore General Hospital, Singapore, Singapore; 3 Division of Nursing, Singapore General Hospital, Singapore, Singapore

## Abstract

**Background:**

Hospital-attributable central line-associated bloodstream infections (HA-CLABSI) are associated with severe patient outcomes. Published data on HA-CLABSI epidemiology in hospitals locally remains limited. This study aimed to determine the HA-CLABSI incidence and risk factors to inform targeted infection prevention practices.

**Methods:**

Retrospective, nested case-control study was performed at Singapore General Hospital from January 2018 to December 2020, involving 127 cases and 252 controls. HA-CLABSI cases developed CLABSI ≥ 3 calendar days of hospitalization. Controls had central line inserted but did not develop CLABSI. Cases and controls were matched on 1:2 ratio for central line insertion date. Multivariable conditional logistic regression was performed to identify independent risk factors for HA-CLABSI, with adjusted odds ratio (aOR), 95% confidence intervals (CI) and *p*-values reported. Variables with *p*-value < 0.05 were statistically significant. HA-CLABSI incidence rate was calculated per 1,000 central line-days.

**Results:**

HA-CLABSI incidence rate during the study period was 8.4/1,000 central line-days. Independent risk factors for HA-CLABSI were transfer to high-risk areas (aOR: 2.03, 95% CI: 1.05–3.92), immunocompromised health status (aOR: 4.62, 95% CI: 2.20–9.69), antibiotic administration (aOR: 7.41, 95% CI: 3.24–16.92), and total parenteral nutrition (aOR: 3.61, 95% CI: 1.49–8.77) being included as indications for central line insertion, insertion of PICC (aOR: 13.61, 95% CI: 3.12–55.53), presence of non-tunneled central lines (aOR: 2.95, 95% CI: 1.48–5.87) and prior MRSA acquisition (aOR: 3.41, 95% CI: 1.83–6.35).

**Conclusion:**

HA-CLABSI remains a significant concern despite on-going infection prevention efforts. Risk factors identified facilitate development of targeted, evidence-based interventions.

## Introduction

Central line utilization continues to rise in tandem with an increasingly complex healthcare landscape. They are life-sustaining in patients requiring hemodialysis, intravenous medications, and parenteral nutrition.^1^ Although crucial for delivery of care in acute and chronic settings, central lines increase risk of healthcare-associated infections (HAI).

Central line-associated bloodstream infections (CLABSI) are a major concern globally as they are associated with severe complications, prolonged hospitalization, increased mortality, and healthcare cost.^2^ CLABSI rates differ between healthcare institutions due to variations in patient characteristics, surveillance definitions, prevention protocols, adherence to infection prevention measures, staff-to-patient ratio, and range of medical services provided.^2^ International Nosocomial Infection Control Consortium (INICC) reported a pooled CLABSI rate of 5.05 per 1,000 central line-days between January 2012 to December 2017, using data from medical-surgical intensive care units (ICUs) in 45 countries in Latin America, Europe, Eastern Mediterranean, Southeast Asia, and Western Pacific.^3^ A multicenter study involving ICUs in three Malaysian hospitals revealed a CLABSI incidence rate (IR) of 4.16 per 1,000 central line-days.^1^ A 2012 meta-analysis estimated the annual cost of CLABSI at USD45,814 per-case.^4^ The United States (US) Centers for Disease Control and Prevention considers CLABSI as a largely preventable HAI.^5^ and recommends implementation of comprehensive prevention bundles to reduce its incidence.^6^

Establishing robust surveillance methods is essential for estimating baseline CLABSI incidence, identifying associated risk factors and guiding infection prevention strategies.^7^ Locally, device-associated and post-operative HAIs in ICUs are reportable to the Ministry of Health, Singapore,^8^ however information on hospital-wide central line utilization, CLABSI incidence, risk factors and causative pathogens remains limited due to a small number of epidemiological and cost-analysis publications in the region.^9^

This study focusses on hospital-attributable CLABSIs (HA-CLABSI) involving all inpatients. Existing literature on HA-CLABSI largely focusses on ICUs and immunocompromised patients due to high central lines utilization and infection risks in these groups. Nevertheless, CLABSI can also occur in other settings underpinning the heterogeneity of the population for which data is lacking. For instance, the INICC reports CLABSI rates primarily from ICUs,^3^ potentially underestimating the burden in non-ICU settings. However, with advances in medical care, and rise in aging population with chronic conditions of varying acuity, several studies have reported an increase in central line utilization and CLABSI incidence in non-ICU settings.^10–13^ As more patients transition to community care following discharge with central lines, HA-CLABSI remains an important surveillance measure that can guide interventions in hospitals.

This study aimed to determine hospital-wide HA-CLABSI incidence and risk factors at a large tertiary hospital in Singapore. By including all inpatient areas (ICUs and general wards), this study offers a comprehensive understanding of HA-CLABSI epidemiology and supports implementation of targeted infection prevention strategies.

## Methods

### Setting

The study was conducted in Singapore General Hospital (SGH), a 1,939 bedded tertiary acute-care hospital with 63 ICU beds, active burns, hematology, oncology and solid organ transplant services, and national cardiothoracic and cancer care centers within its premises.

### Study design

This was a single-center, retrospective case-control study, nested within a cohort of adult patients aged ≥18 years and had ≥1 central lines inserted for at least 48hours from admission date. The study included data from January 2018 to December 2020. Nested case-control studies employs the case-control methodology within a well-defined cohort (here, all patients with central lines inserted), wherein cases and controls are matched at the time of event. This design is suitable when time of exposure is of importance.^14^ The Coronavirus Disease 2019 (COVID-19) pandemic significantly impacted hospital operations and infection prevention practices.^15^ Therefore, compliance with best practices may have been affected due to competing clinical and operation priorities, thereby influencing central lines care and consequently increasing HA-CLABSI risk.^16^ Temporal matching for central line insertion date was important to mitigate the potential confounding effects of pandemic related infection prevention changes, and to ensure that cases and controls were exposed to similar risk environment and infection prevention practices.

### HA-CLABSI Definition

CLABSI was determined based on the US National Healthcare Safety Network (NHSN) guidelines.^17^ A patient was considered to have CLABSI, if they had a central line inserted and met one of the following criteria— (i) a positive blood culture with a recognized bacterial or fungal pathogen not considered as common commensal by the NHSN; (ii) same NHSN-defined common commensal identified from a blood culture with two or more blood specimens drawn on separate occasions, in the presence of at least one of the following symptoms— fever (≥38 °C), chills, or hypotension; (iii) organisms identified in the blood culture were not attributable to infection at another body site.^17^ HA-CLABSI was defined as CLABSI event occurring on or after 3rd calendar day of hospital admission.

## Patients

All adult inpatients who met the NHSN criteria for HA-CLABSI during the study period were included as cases. Controls were selected from a pool of inpatients with at least one central line inserted but did not develop CLABSI throughout their hospitalization or end of study period, whichever occurred first. Cases and controls were matched on a 1:2 ratio based on insertion date, wherein controls with central lines inserted ±30days of their corresponding case’s insertion date were included. If multiple controls met the matching criteria, the two with central line insertion dates closest to that of the corresponding case were selected.

### Data collection

Demographic, clinical, and central line-related variables were selected based on existing literature and biological plausibility. Data was collected through review of electronic medical records. Clinical data collection was anchored on a pre-defined at-risk window. At-risk period for cases was defined from central line insertion date to positive blood culture date (i.e., HA-CLABSI diagnosis date). For controls, at-risk period extended from central line insertion date to the corresponding case’s HA-CLABSI date or control’s discharge date or study period end, whichever occurred first (referred to as censoring date for controls).

Demographic variables included age at admission, and sex. Clinical variables included mobility status, basal metabolic index, underlying comorbidities, neutropenia (absolute neutrophil count < 1,500 cells/mm^3^), transfer to high-risk areas (ICU, intermediate care area [ICA] and high-dependency [HD] units) spanning up to 14 days prior to start of at-risk period, acquisition of multidrug-resistant organisms (MDRO) in the same admission prior to central line insertion, and prior bloodstream infection within four weeks preceding central line insertion. Indications for central line insertion included chemotherapy, hemodialysis, total parenteral nutrition (TPN), prior surgery, and administration of antibiotics and immunosuppressants. Patients were classified as having dependent mobility status if admission nursing documentation described them as “wheelchair-bound,” “bed-bound,” or “requiring assistance with mobility.” Patients with end-stage renal failure requiring hemodialysis, history of organ transplant, hematologic malignancy, or administration of chemotherapy or immunosuppressants during at-risk period were considered immunocompromised. Underlying comorbidities were identified using the International Classification of Diseases– Tenth Revision, Australian Modification (ICD-10-AM) diagnostic codes. Central line-related variables included type of central line (central venous catheter [CVC], peripherally inserted central catheter [PICC], port-a-cath, vascular catheter), number of lumens and insertion site (brachial, jugular, subclavian, femoral, basilic). MDROs included methicillin-resistant *Staphylococcus aureus* (MRSA), carbapenemase-producing *Enterobacteriaceae* (CPE) and vancomycin-resistant *Enterococci* (VRE). Inpatient mortality was defined as death occurring in the same hospitalization episode, after HA-CLABSI diagnosis for cases and after censor date for controls.

### Statistical analysis

Statistical analysis was performed in R version 4.0.1.^18^ Categorical data were summarized as counts and percentages. Continuous variables were evaluated for normality using Kolmogorov-Smirnov test and histograms and presented as median and interquartile range (IQR). Comorbidities with biological plausibility of association with HA-CLABSI and sufficient sample size were included in bivariate analysis. Conditional logistic regression was used for bivariate analysis between outcome (case-control status) and individual variables. Unadjusted odds ratios (OR) with their corresponding 95% confidence intervals (CI) and *p*-values were recorded. Variables with biological plausibility and *p*-values < 0.05 in the bivariate analysis were included in the multivariable regression. Collinearity of these variables was assessed using Pearson’s correlation coefficient. Backward stepwise multivariable conditional logistic regression was performed to identify independent risk factors for HA-CLABSI and their corresponding adjusted ORs (aOR), 95% CI and *p*-values were noted. Variables with *p*-values < 0.05 were considered statistically significant. At each step of multivariable regression, variables were removed based on their highest *p*-value. Model fit was assessed using Akaike Information Criterion and Nagelkerke *R* ^2^. HA-CLABSI IR was expressed per 1,000 central line-days.

### Ethics

A waiver of consent was obtained from the SingHealth Centralized Institutional Review Board (CIRB Ref: 2020/2799) as this study was performed as part of infection prevention efforts in the hospital.

## Results

127 HA-CLABSI cases and 252 controls were included in the study. HA-CLABSI IR between January 2018 to December 2020 was 8.4 per 1,000 central line-days.

Table 1 summarizes the patient and central line-related factors. Variables significantly associated with HA-CLABSI in the bivariate analysis were immunocompromised health status (OR: 1.68, 95% CI: 1.00–2.80), bloodstream infection in the four weeks preceding central line insertion (OR: 2.13, 95% CI: 1.13–4.03), transfer to high-risk areas (OR: 2.13, 95% CI: 1.29–3.50), clinical indications for central line insertion including antibiotic administration (OR: 4.74, 95% CI: 2.61–8.63), and TPN (OR: 2.39, 95% CI: 1.24–4.62), insertion of PICC (OR: 9.91, 95% CI: 2.91–33.77) and non-tunneled central line (OR: 3.12, 95% CI: 1.75–5.57) and prior acquisition of MRSA (OR: 2.74, 95% CI: 1.70–4.42), VRE (OR: 2.19, 95% CI: 1.36–3.55), and CPE (OR: 2.21, 95% CI: 1.31–3.37) (Table 1). There was no correlation observed between these variables.

**Table 1.** Patient demographic and clinical characteristics and central line-related factors

	Case *n* = 127	Control *n* = 252	Unadjusted OR	95%CI	*p*-value*
**Demographics**					
Sex					
Male, (%)	79 (62.2)	140 (55.6)	0.75	0.47–1.19	0.221
Age at admission (years), median (IQR)	64 (55–72)	64 (56–72)	0.99	0.98–1.01	0.386
**Clinical characteristics** (**%)**					
Dependent mobility status^a^	40 (31.5)	72 (28.6)	0.85	0.52–1.40	0.524
BMI	25.2 (6.4)	24.1 (5.6)	1.04	1.00–1.08	0.057
Immunocompromised health status^b^	61 (48.0)	98 (38.9)	1.68	1.00–2.80	0.049
Neutropenia^c^	29 (22.8)	36 (14.3)	1.84	1.04–3.26	0.036
Underlying Comorbidities (%)					
Diabetes mellitus type II	63 (49.6)	119 (47.2)	1.11	0.71–1.73	0.650
Cancer	30 (23.6)	68 (27.0)	0.84	0.51–1.37	0.480
Dementia	2 (1.6)	1 (0.4)	4.00	0.36–44.11	0.258
Burns/TEN	5 (3.9)	7 (2.8)	2.00	0.40–9.91	0.396
Prior bloodstream infections^d^	21 (16.5)	21 (8.3)	2.13	1.13–4.03	0.020
Transfer to high-risk areas^e^ (%)	93 (73.2)	147 (58.3)	2.13	1.29–3.50	0.003
Indications for central line insertion (%)					
Antibiotic administration	44 (34.6)	29 (39.7)	4.74	2.61–8.63	< 0.001
Chemotherapy	15 (11.8)	18 (7.1)	1.81	0.86–3.81	0.120
Immunosuppressant medications	23 (18.1)	35 (13.9)	1.38	0.77–2.49	0.281
Total parenteral nutrition	22 (17.3)	21 (8.3)	2.39	1.24–4.62	0.009
Hemodialysis	57 (44.9)	94 (37.3)	1.71	0.97–3.01	0.065
Surgery prior to central line insertion	55 (43.3)	94 (37.3)	1.39	0.85–2.28	0.192
**Central line-related factors** (**%)**					
Duration at-risk (Days), median (IQR)	15 (31)	6 (9)	–	–	–
Central line with ≥3 lumens	45 (35.4)	96 (38.1)	0.84	0.49–1.43	0.524
Type of central line					
PICC^f^	47 (37.0)	63 (25.0)	9.91	2.91–33.77	< 0.001
Non-tunneled central line^g^	94 (74.0)	142 (56.3)	3.12	1.75–5.57	< 0.001
Elective insertion	108 (85.0)	207 (82.1)	1.33	0.68–2.61	0.404
Insertion site- femoral vein	16 (12.6)	23 (9.1)	1.51	0.72–3.17	0.276
**Prior MDRO acquisition** ^**h**^ **(%)**					
MRSA	62 (16.4)	68 (27.0)	2.74	1.70–4.42	< 0.001
VRE	47 (37.0)	54 (21.4)	2.19	1.36–3.55	0.001
CPE	33 (26.0)	33 (13.1)	2.21	1.31–3.37	0.003
**Complications** (**%)**					
Inpatient mortality	32 (25.2)	35 (13.9)	2.02	1.19–3.43	0.009

Note. CI, confidence interval. IQR, interquartile range. BMI, body mass index. TEN, toxic epidermal necrolysis. MDRO, multidrug-resistant organisms. MRSA, Methicillin-Resistant *Staphylococcus aureus*; VRE, Vancomycin-resistant *Enterococcus*. CPE, Carbapenemase-producing *Enterobacteriaceae*.

Categorical data are shown as number (%), continuous data are shown in mean (SD) or median (IQR).

* Variables with *p*-value < 0.05 were considered statistically significant.

^a^Mobility data was collected as of the admission notes. Dependent mobility was defined as “wheelchair bound, “bed-bound” or “requiring assistance” status.

^b^Patients with end-stage renal failure requiring hemodialysis and/or a history of transplant/hematological malignancy and/or receiving chemotherapy or immunosuppressants during the at-risk period.

^c^Absolute neutrophil count < 100 cells/mm^3^.

^d^Any bloodstream infections in the four weeks preceding central line insertion for cases and controls.

^e^Transfer to any high-risk areas (i.e., intensive care unit, intermediate care area, high-dependency unit) within ±2 weeks of central line insertion.

^f^Other central lines (i.e., central venous catheter, port-a-cath, and vascular catheter) were used as the reference level for PICC.

^g^Tunneled central line was used as the reference level for non-tunneled central line.

^h^Any MDRO acquired in the same admission, prior to central line insertion.

Transfer to high-risk areas (aOR: 2.03, 95% CI: 1.05–3.92), immunocompromised health status (aOR: 4.62, 95% CI: 2.20–9.69), clinical indications for central line insertion including antibiotic administration (aOR: 7.41, 95% CI: 3.24–16.92), and TPN (aOR: 3.61, 95% CI: 1.49–8.77), insertion of PICC (aOR: 13.61, 95% CI: 3.12–55.53) and non-tunneled central lines (aOR: 2.95, 95% CI: 1.48–5.87) and prior MRSA acquisition (aOR: 3.41, 95% CI: 1.83–6.35) were identified as statistically significant risk factors for HA-CLABSI (Table 2).

**Table 2.** Independent risk factors significantly associated with HA-CLABSI

	**aOR**	**95%CI**	***p*** **-value** ^*^
**Clinical characteristics**			
Transfer to high-risk areas^a^	2.03	1.05–3.92	0.035
Underlying comorbidities			
Immunocompromised health status	4.62	2.20–9.69	<0.001
**Indications for central line insertion**			
Antibiotic administration	7.41	3.24–16.92	< 0.001
Total parenteral nutrition	3.61	1.49–8.77	0.005
**Central Line related factors**			
Type of central line			
PICC^b^	13.61	3.12–55.53	< 0.001
Non-tunneled central line^c^	2.95	1.48–5.87	< 0.001
**Prior MDRO acquisition**			
MRSA^d^	3.41	1.83–6.35	< 0.001

Note. aOR, adjusted odds ratio, PICC, peripherally inserted central catheter. MDRO, multidrug-resistant organism. Methicillin-Resistant *Staphylococcus aureus*.

* *p*-value < 0.05 was considered significant in the backward stepwise multivariable conditional logistic regression.

^a^Transfer to high-risk areas (i.e., intensive care unit, intermediate care area, high-dependency unit) within ±2 weeks of central line insertion.

^b^Other central lines (i.e., central venous catheter, port-a-cath, and vascular catheter) were used as the reference level for PICC.

^c^ Tunneled central line was used as the reference level for non-tunneled central line.

^d^MRSA acquired in the same admission, prior to central line insertion.

Table 3 describes the distribution of microorganisms causing HA-CLABSI. A total of 172 pathogens were isolated from 127 cases. Leading pathogens for HA-CLABSI were gram-negative bacilli (41.9%), followed by gram-positive bacteria (27.3%), fungi (15.1%) and MDROs (12.8%). Most common gram-negative bacilli were *Klebsiella pneumoniae* (9.3%), *Pseudomonas aeruginosa* (6.4%), *Enterobacter cloacae* complex (5.8%), *Stenotrophomonas maltophilia* (4.7%), and *Acinetobacter* spp. (4%). *Staphylococcus aureus* (8.4%), and *Staphylococcus epidermidis* (4.7%) were the predominant gram-positive bacteria. *Candida parapsilosis* complex (5.2%) and *Candida glabrata* complex (4.7%) were the most common causative fungi. Total MDRO burden was 12.8%, of which MRSA (7.6%), and VRE (4.1%) were common causative organisms.

**Table 3.** Distribution of microorganisms isolated from HA-CLABSI cases

**Microorganisms**	**Number (%) of microorganisms** (*n* = 172) *^
**Gram-negative bacteria**	72 (41.9)
*Enterobacterales*	
* Klebsiella pneumoniae*	16 (9.3)
* Enterobacter cloacae* complex	10 (5.8)
Other *Enterobacteriaceae spp.* ^a^	15 (8.7)
*Pseudomonas* species	
* Pseudomonas aeruginosa*	11 (6.4)
* Pseudomonas spp.* ^*b*^	2 (1.2)
*Stenotrophomonas maltophilia*	8 (4.7)
*Acinetobacter spp.*	7 (4.1)
*Elizabethkingia spp.*	5 (2.9)
*Achromobacter spp.*	2 (1.2)
*Bacteroides fragilis*	2 (1.2)
*Chryseobacterium spp.*	2 (1.2)
Other gram negative spp.^c^	3 (1.7)
**Gram-positive bacteria**	47 (27.3)
*Staphylococcus* species	
* Staphylococcus aureus*	14 (8.1)
* Staphylococcus epidermidis*	8 (4.7)
Other *Staphylococcus spp.* ^d^	8 (4.7)
*Enterococcus spp.*	6 (3.5)
*Streptococcus spp.*	2 (1.2)
*Corynebacterium spp.*	1 (0.6)
Other gram-positive spp.^e^	2 (1.2)
**MDRO**	22 (12.8)
MRSA	13 (7.6)
VRE	7 (4.1)
CPE (OXA-48 genotype)	2 (1.2)
**Fungus**	26 (15.1)
*Candida* spp.	
* Candida parapsilosis* complex	9 (5.2)
* Candida glabrata* complex*/Candida glabrata*	8 (4.7)
* Candida auris*	2 (1.2)
* Candida tropicalis*	2 (1.2)
* Candida albicans*	1 (0.6)
* Candida dubliniensis*	1 (0.6)
* Candida haemulonis* complex	1 (0.6)
*Trichosporon* species	
* Trichosporon asahii*	1 (0.6)
* Trichosporon japonicum*	1 (0.6)

Note. MDRO, multidrug-resistant organism. spp., species. MRSA, methicillin-resistant *Staphylococcus aureus.* VRE, Vancomycin-resistant *enterococcus*. CPE, Carbapenemase-producing *Enterobacteriaceae.*

* The total number of microorganisms exceeds the total number of CLABSI cases (*n* = 127).

^The percentages are calculated out of 172.

^a^Other *Enterobacteriaceae spp.* included one *Citrobacter koseri*, five *Escherichia coli*, three *Klebsiella aerogenes*, one *Proteus mirabilis*, and five *Serratia marcescens*.

^b^Other *Pseudomonas spp.* included one *Pseudomonas putida* and one unidentified *Pseudomonas spp.*

^c^Other gram-negative spp. included one *Morganella morganii*, one *Rothia mucilaginosa* and one unidentified *Delftia spp*.

^d^Other *Staphylococcus spp.* included three coagulase negative *Staphylococcus*, two *Staphylococcus capitis*, three *Staphylococcus hemolyticus* and eight *Staphylococcus epidermidis*.

^e^Other gram-positive spp. included one *Leuconostoc* spp. and one unidentified bacillus spp.

## Discussion

Understanding the burden and risk factors can enable risk stratification of patients and development of targeted strategies for early identification and prevention of HA-CLABSI. Despite on-going infection prevention efforts, several studies report significant CLABSI incidence in their settings.^19–21^ In a retrospective cohort study in 2020, Baier et al reported an IR of 10.6 per 1,000 central venous catheter-days and prevalence of 18.2%, from 610 hematologic-oncologic patients.^19^ In a nationwide study of acute-care hospitals in US from 2011 to 2017, Novosad et al reported 59,461 CLABSIs from adult ICUs and 40,763 from adult wards to the NHSN,^20^ thereby highlighting the need for robust HA-CLABSI surveillance beyond critical-care areas. Mishra et al reported an IR of 17.04 per 1,000 catheter-days in a medical ICU over a 16-month period in an Indian tertiary hospital.^21^ Our hospital-wide HA-CLABSI IR was 8.4 per 1,000 central line-days.

The known risk factors for HA-CLABSI include older age, underlying comorbidities, higher Acute Physiology and Chronic Health Evaluation (APACHE) score, neutropenia, administration of chemotherapy, immunosuppressants, parenteral nutrition and antibiotics, immunocompromised health status, hematological malignancies, prior surgery, insertion of other invasive devices, prolonged hospitalization and catheterization duration before CLABSI acquisition, prior ICU stay, multi-lumen central lines, concurrent use of ≥1central lines, non-tunneled central lines, position of central line, location of insertion and central line maintenance.^1,12,19,21–23^ Risk factors significantly associated with HA-CLABSI in our study were transfer to high-risk areas, immunocompromised health status, administration of antibiotics and TPN via central lines, insertion of non-tunneled central line and PICC and prior MRSA acquisition. These findings are aligned with existing literature. A recent multicenter study by Rajandra et al. involving three Malaysian hospitals found that patients with extended ICU stay were at almost two-fold higher odds of CLABSI.^1^ Mishra et al reported that immunocompromised patients were at 10.5 times higher odds of developing CLABSI.^21^ Ippolito et al demonstrated that patients receiving TPN were at 4.33 times higher odds of developing CLABSI.^24^ Patients with prior ICU stays, immunocompromised health status and requiring administration of TPN and antibiotics are vulnerable to HAIs, such as HA-CLABSI, due to their severity of illness, multiple comorbidities, prolonged hospitalization, frequent clinical interventions, emergency insertions and utilization of multiple central lines.^1,10,23^ These provide additional opportunities for compromising sterile barriers and enabling migration of microorganisms from patient’s skin and external surfaces to internal luminal surface of central lines, resulting in biofilms formation.^25,26^ The high nutritional content of TPN can also promote intraluminal biofilm formation, which often harbors microorganisms that are drug-resistant and evade host immune responses. Catheter hubs are often primary entry sites for microorganisms and biofilm formation.^26^ Therefore, infection prevention measures are crucial to prevent biofilm formation and pathogen colonization in central lines. Malnutrition in hospitalized patients can be exacerbated by dextrose infusion in TPN, leading to hyperglycemia and consequent suppression of patient’s immune system as a stress-response, thereby increasing susceptibility to infections such as HA-CLABSI.^26^

Baier et al found the prevalence of CLABSI to be 18% in patients with non-implanted central lines.^19^ Non-tunneled catheters are temporary central lines that are inserted percutaneously and, therefore, account for majority of CLABSIs.^25^ In a prospective surveillance study, Templeton et al demonstrated that each additional lumen was associated with 4.4 times higher hazards of developing CLABSI.^27^ However, in our study, utilization of multi-lumen central lines was not associated with HA-CLABSI.

The optimal site for central line insertion remains debatable. According to the Asia Pacific Society of Infection Control guidelines for CLABSI prevention, femoral catheters should be avoided due to higher risk of infections and deep vein thrombosis.^28^ Merrer et al demonstrated that femoral catheterization was associated with a significantly increased risk of CLABSI compared to subclavian site (19.8% vs 4.5%; IR of 20 vs 3.7 per 1,000 catheter-days).^29^ This could be due to the relatively high concentration of skin flora in the groin region.^6^ However, Marik et al in 2012 and Timsit et al in 2013 reported a comparable risk of infection between the three typical insertion sites (subclavian, jugular, and femoral vein).^30,31^ We found no significant association between femoral site insertion for central lines and HA-CLABSI in our study.

HA-CLABSI is associated with increased risk of complications. MDROs add to the existing complexity of clinical management of HA-CLABSI.^10^ In this study, MRSA acquisition prior to central line insertion was significantly associated with HA-CLABI risk. MDROs (i.e., MRSA, VRE and CPE) accounted for 12.8% of the causative organisms identified. Predominant species causing HA-CLABSI were gram-negative bacilli followed by gram-positive bacteria and fungi. *Enterobacterales* species was the most common organism (14.5% of all isolates). Other settings have also revealed a shift in the dynamic hospital ecosystem from gram-positive to gram-negative bacteria.^10,20,32–35^ Therefore, addressing pathogen profile, prevalence and antibiograms may offer insights into hospital bioburden, support clinical decision-making and enable development of focused infection prevention measures such as screening and decolonization protocols, surveillance methods and antimicrobial stewardship programs.

The risk factors identified in this study were largely modifiable, indicating HA-CLABSI in our setting may be preventable. Local and international guidelines recommend several evidence-based HA-CLABSI prevention strategies including, standardized central line insertion and removal checklists, aseptic technique, maximal barrier precautions, chlorhexidine-impregnated dressings, routine review of central line necessity to reduce dwell time, disinfection of catheter hubs, needless connectors, and injection ports before accessing central line, selecting tunneled central lines when long-term utilization is anticipated, staff training, care-giver education, and compliance audits.^6,28,36,37^ HA-CLABSI surveillance in ICU and non-ICU settings is critical for identifying trends and reducing incidence. Given the significant association with TPN and antibiotic administration, judicious utilization of these interventions is warranted with regular assessments to discontinue when no longer necessary.^38,39^ Furthermore, the significant association between prior MRSA acquisition and CLABSI supports importance of contact precautions, hand hygiene and consideration of antiseptic or antimicrobial impregnated central lines, and antimicrobial lock therapy for long-term central lines in mitigating HA-CLABSI risk.^28,36^ Emerging approaches such as individualized risk prediction scores may enable early identification of patients at risk of HA-CLABSI.^40^

This study has several strengths. First, broad inclusion of hospital-wide HA-CLABSI patients enables a more comprehensive understanding of the risk across diverse inpatient settings where HA-CLABSI remains under-studied. Second, utilization of standardized NHSN definitions ensures consistency with international surveillance criteria and enables comparability of the findings. Third, temporal matching of cases and controls allows mitigation of any confounding effects due to pandemic-related changes in HA-CLABSI prevention practices, ensuring that both cases and controls were exposed to a similar risk environment. Lastly, being one of the largest tertiary-level hospitals, SGH receives a diverse patient population, therefore findings of this study may offer regional and local insights into HA-CLABSI epidemiology.

There were some limitations of this study. First, data collection relied on review of electronic medical records, which may introduce information bias due to potential inaccuracies in documentation, particularly for central line insertion and removal dates. Second, since this was a single-center study, the findings may have limited generalizability to other healthcare settings. Third, we did not analyze compliance rates and impact of our interventional bundles on HA-CLABSI incidence. Finally, residual confounding due to unmeasured variables cannot be excluded.

In conclusion, despite implementation of prevention bundles, HA-CLABSI remains of significant concern. Extending surveillance and prevention beyond ICU settings and understanding local epidemiology can guide tailored infection prevention measures.

## References

[1] Rajandra A , Yunos NM , Teo CH , et al. Incidence, compliance, and risk factor associated with central line-associated bloodstream infection (CLABSI) in intensive care unit (ICU) patients: A multicenter study in an upper middle-income country. Antibiotics 2025;14:271. 40149082 10.3390/antibiotics14030271 PMC11939773

[2] Al-Khawaja S , Saeed NK , Al-khawaja S , Azzam N , Al-Biltagi M. Trends of central line-associated bloodstream infections in the intensive care unit in the Kingdom of Bahrain: Four years’ experience. World J Crit Care Med 2021;10:220–231. 34616658 10.5492/wjccm.v10.i5.220 PMC8462019

[3] Rosenthal VD , Bat-Erdene I , Gupta D , et al. International nosocomial infection control consortium (INICC) report, data summary of 45 countries for 2012-2017: Device-associated module. Am J Infect Control 2020;48:423–432. 31676155 10.1016/j.ajic.2019.08.023

[4] Zimlichman E , Henderson D , Tamir O , et al. Health care-associated infections: A meta-analysis of costs and financial impact on the US health care system. JAMA Intern Med 2013;173:2039–2046. 23999949 10.1001/jamainternmed.2013.9763

[5] Central Line-associated Bloodstream Infection (CLABSI) Basics [Internet]. Centers for Disease Control and Prevention, 2024. Available from: https://www.cdc.gov/clabsi/about/index.html

[6] O’Grady NP , Alexander M , Burns LA , et al. (PDF) Guidelines for the prevention of intravascular catheter-related infections [Internet], 2024. Available from: https://www.cdc.gov/infection-control/hcp/intravascular-catheter-related-infection/index.html

[7] Cai Y , Venkatachalam I , Tee NW , et al. Prevalence of healthcare-associated infections and antimicrobial use among adult inpatients in Singapore acute-care hospitals: Results from the first national point prevalence survey. Clin Infect Dis 2017;64(suppl_2):S61–S67. 28475790 10.1093/cid/cix103

[8] The National Infection Prevention and Control Guidelines for Acute Healthcare Facilities [Internet]. Ministry of Health Singapore, 2024 [cited 2017]. https://www.hpp.moh.gov.sg/all-healthcare-professionals/guidelines/GuidelineDetails/infection-prevention-and-control-guidelines-and-standards

[9] Ling ML , Apisarnthanarak A , Madriaga G. The burden of healthcare-associated infections in Southeast Asia: A systematic literature review and meta-analysis. Clin Infect Dis 2015;60:1690–1699. 25676799 10.1093/cid/civ095

[10] Alwazzeh MJ , Alnimr A , Al Nassri SA , et al. Microbiological trends and mortality risk factors of central line-associated bloodstream infections in an academic medical center 2015–2020. Antimicrob Resist Infect Control 2023;12:128. 37981696 10.1186/s13756-023-01338-5 PMC10659071

[11] Rhee Y , Heung M , Chen B , Chenoweth CE. Central line-associated bloodstream infections in non-ICU inpatient wards: A 2-year analysis. Infect Control Hosp Epidemiol 2015;36:424–430. 25782897 10.1017/ice.2014.86

[12] Seo HK , Hwang JH , Shin MJ , et al. Two-year hospital-wide surveillance of central line-associated bloodstream infections in a Korean hospital. J Korean Med Sci 2018;33:e280. 30402047 10.3346/jkms.2018.33.e280 PMC6209765

[13] Climo M , Diekema D , Warren DK , et al. Prevalence of the use of central venous access devices within and outside of the intensive care unit: Results of a survey among hospitals in the prevention epicenter program of the centers for disease control and prevention. Infect Control Hosp Epidemiol 2003;24:942–945. 14700410 10.1086/502163

[14] Partlett C , Hall NJ , Leaf A , Juszczak E , Linsell L. Application of the matched nested case-control design to the secondary analysis of Trial Data. BMC Med Res Methodol 2020;20:117. 32410578 10.1186/s12874-020-01007-w PMC7227268

[15] Wee LEI , Sim XYJ , Conceicao EP , et al. Containing covid-19 outside the Isolation Ward: The impact of an infection control bundle on environmental contamination and transmission in a cohorted general Ward. Am J Infect Control 2020;48:1056–1061. 32599101 10.1016/j.ajic.2020.06.188 PMC7319619

[16] Satta G , Rawson TM , Moore LSP. Coronavirus disease 2019 (covid-19) impact on central-line-associated bloodstream infections (CLABSI): A systematic review. Infect Prev Pract 2023;5:100313. 37920796 10.1016/j.infpip.2023.100313 PMC10618700

[17] Bloodstream Infection Event (Central Line-Associated Bloodstream Infection and Non-central Line Associated Bloodstream Infection) [Internet]. Centers for Disease Control and Prevention, 2024. https://www.cdc.gov/nhsn/psc/bsi/index.html

[18] R Core Team. R: A Language and Environment for Statistical Computing. R Foundation for Statistical Computing, Vienna, Austria; 2020 URL: https://www.R-project.org/

[19] Baier C , Linke L , Eder M , et al. Incidence, risk factors and healthcare costs of central line-associated nosocomial bloodstream infections in hematologic and oncologic patients. PLoS ONE 2020;15:e0227772. 31978169 10.1371/journal.pone.0227772 PMC6980604

[20] Novosad SA , Fike L , Dudeck MA , et al. Pathogens causing central-line–associated bloodstream infections in acute-care hospitals—United States, 2011–2017. Infect Control Hosp Epidemiol 2020;41:313–319. 31915083 10.1017/ice.2019.303 PMC13108540

[21] Mishra SB , Misra R , Azim A , et al. Incidence, risk factors and associated mortality of central line-associated bloodstream infections at an intensive care unit in Northern India. Int J Qual Health Care 2017;29:63–67. 27940521 10.1093/intqhc/mzw144

[22] Huang H , Chang Q , Zhou Y , Liao L. Risk factors of central catheter bloodstream infections in intensive care units: A systematic review and meta-analysis. PLOS ONE 2024;19:e0296723. 38652718 10.1371/journal.pone.0296723 PMC11037535

[23] Moriyama K , Ando T , Kotani M , et al. Risk factors associated with increased incidences of catheter-related bloodstream infection. Medicine 2022;101:e31160. 36281147 10.1097/MD.0000000000031160 PMC9592381

[24] Ippolito P , Larson EL , Furuya EY , Liu J , Seres DS. Utility of electronic medical records to assess the relationship between parenteral nutrition and Central line–associated bloodstream infections in adult hospitalized patients. J Parenter Enteral Nutr 2014;39:929–934. 10.1177/0148607114536580 PMC4256122 24898208

[25] Haddadin Y. Central line–associated blood stream infections [Internet]. U.S. National Library of Medicine, 2022. https://www.ncbi.nlm.nih.gov/books/NBK430891/

[26] Opilla M. Epidemiology of bloodstream infection associated with parenteral nutrition. Am J Infect Control 2008;36:S173–e5. 10.1016/j.ajic.2008.10.007 19084152

[27] Templeton A , Schlegel M , Fleisch F , et al. Multilumen central venous catheters increase risk for catheter-related bloodstream infection: Prospective surveillance study. Infection 2008;36:322–327. 18663408 10.1007/s15010-008-7314-x

[28] Ling ML , Apisarnthanarak A , Jaggi N , et al. APSIC guide for prevention of central line associated bloodstream infections (CLABSI). Antimicrob Resist Infect Control 2016;5:16. 27152193 10.1186/s13756-016-0116-5 PMC4857414

[29] Merrer J , De Jonghe B , Golliot F. Complications of femoral and subclavian venous catheterization in critically ill patients: A randomized controlled trial. JAMA 2001;286:700–707. 11495620 10.1001/jama.286.6.700

[30] Marik PE , Flemmer M , Harrison W. The risk of catheter-related bloodstream infection with femoral venous catheters as compared to subclavian and internal jugular venous catheters. Crit. Care Med 2012;40:2479–2485. 22809915 10.1097/CCM.0b013e318255d9bc

[31] Timsit JF , Bouadma L , Mimoz O , et al. Jugular versus femoral short-term catheterization and risk of infection in intensive care unit patients. Causal analysis of two randomized trials. Am J Respir Crit Care Med 2013;188:1232–1239. 24127770 10.1164/rccm.201303-0460OC

[32] Kale P , Khodare A , Pindi G , Joy L , Khillan V. Incidence, microbiological profile, and impact of preventive measures on central line-associated bloodstream infection in liver care intensive care unit. Indian J Crit Care Med 2019;24:17–22. 10.5005/jp-journals-10071-23325 PMC7050171 32148344

[33] Kuo SH , Lin WR , Lin JY , et al. The epidemiology, antibiograms and predictors of mortality among critically-ill patients with central line-associated bloodstream infections. J Microbiol Immunol Infect 2018;51:401–410. 28943144 10.1016/j.jmii.2017.08.016

[34] Chaftari AM , Hachem R , Jiang Y , Shah P , Hussain A , Hamal ZA , et al. Changing epidemiology of catheter-related bloodstream infections in cancer patients. Infect Control Hosp Epidemiol 2018;39:727–729. 29770754 10.1017/ice.2018.75

[35] Pitiriga V , Kanellopoulos P , Bakalis I , Kampos E , Sagris I , Saroglou G , et al. Central venous catheter-related bloodstream infection and colonization: The impact of insertion site and distribution of multidrug-resistant pathogens. Antimicrob Resist Infect Control 2020;9:189. 33261661 10.1186/s13756-020-00851-1 PMC7708904

[36] Malek AE , Raad II. Preventing catheter-related infections in cancer patients: A review of current strategies. Expert Rev Anti Infect Ther 2020;18:531–538. 32237923 10.1080/14787210.2020.1750367

[37] Morisi N , Montani M , Ehode EN , et al. Evaluating short-term outcomes of tunneled and non-tunneled central venous catheters in hemodialysis. J Clin Med 2024;13:3664. 38999230 10.3390/jcm13133664 PMC11242506

[38] Lum JH , Yeong HEL , Tan PE , Salazar E , Lee T , Ng YC , et al. Singapore clinical guideline on parenteral nutrition in adult patients in the acute hospital setting. Ann Acad Med Singap 2025;54:350–369. 40637607 10.47102/annals-acadmedsg.2024318

[39] Ahmed SK , Hussein S , Qurbani K , et al. Antimicrobial resistance: Impacts, challenges, and future prospects. J Med Surg Public Health 2024;2:100081.

[40] Herc E , Patel P , Washer LL , Conlon A , Flanders SA , Chopra V. A model to predict central-line–associated bloodstream infection among patients with peripherally inserted central catheters: The MPC score. Infect Control Hosp Epidemiol 2017;38:1155–1166. 28807074 10.1017/ice.2017.167

